# Shear-Viscosity-Dependent Effect of a Gum-Based Thickening Product on the Safety of Swallowing in Older Patients with Severe Oropharyngeal Dysphagia

**DOI:** 10.3390/nu15143279

**Published:** 2023-07-24

**Authors:** Mireia Bolivar-Prados, Yuki Hayakawa, Noemi Tomsen, Viridiana Arreola, Weslania Nascimento, Stephanie Riera, Satomi Kawakami, Kazuhiro Miyaji, Yasuhiro Takeda, Jun Kayashita, Pere Clavé

**Affiliations:** 1 Gastrointestinal Physiology Laboratory, Hospital de Mataró, Universitat Autònoma de Barcelona, 08304 Mataró, Spain; mbolivar@csdm.cat (M.B.-P.); ntomsen@csdm.cat (N.T.);; 2 Centro de Investigación Biomédica en Red de Enfermedades Hepáticas y Digestivas (Ciberehd), 28029 Madrid, Spain; 3 R&D Division, Morinaga Milk Industry Co., Ltd., Zama-City 222-0033, Japan; y-hayakawa@morinagamilk.co.jp (Y.H.); s_kawakm@morinagamilk.co.jp (S.K.); k_miyazi@morinagamilk.co.jp (K.M.); ya_taked@morinagamilk.co.jp (Y.T.); 4 Department of Health Sciences, Faculty of Human Culture and Science, Prefectural University of Hiroshima, Hiroshima 734-8558, Japan; kayashita@pu-hiroshima.ac.jp

**Keywords:** thickening agent, thickening product, shear viscosity, shear rate, salivary amylase, dysphagia

## Abstract

Fluid thickening is a valid therapeutic strategy for patients with oropharyngeal dysphagia (OD). The main aim of this study was to determine the therapeutic effect of the xanthan-gum-based thickener Tsururinko Quickly (TQ, Morinaga Milk Co., Tokyo, Japan) in older patients with severe OD. A total of 85 patients (83.32 ± 6.75 y) with OD and a penetration–aspiration score (PAS) of n ≥ 3 were studied by videofluoroscopy while swallowing duplicate 10 mL boluses at <50 mPa·s, 100, 200, 400, 800, and 1600 mPa·s, to assess the safety and efficacy of swallowing and the biomechanics of a swallowing response at each viscosity level. At <50 mPa·s, only 16.25% patients swallowed safely, 45% had penetrations (PAS 3–5), and 38.75% had aspirations (PAS 6–8). Fluid thickening with TQ greatly increased the prevalence of patients with safe swallowing from 62.90% at 100 mPa·s to 95.24% at 1600 mPa·s in a shear-viscosity-dependent manner. The penetrations and aspirations were significantly reduced to 3.60% and 1.19%, respectively, at 1600 mPa·s. The threshold viscosity was 100 mPa·s and the increasing viscosity above 800 mPa·s did not further improve the therapeutic effect significantly. Increasing the shear viscosity significantly reduced the time to laryngeal vestibule closure (−16.70%), increased the time to upper oesophageal sphincter opening (+26.88%), and reduced the pharyngeal bolus velocity (−31.62%) without affecting the pharyngeal residue. TQ has a strong shear-viscosity-dependent effect on the safety of swallowing in older patients with severe OD without increasing the pharyngeal residue. The therapeutic range for TQ is 100–800 mPa·s, with 200 and 800 mPa·s being the optimal doses to cover the needs of older patients with OD.

## 1. Introduction

Oropharyngeal dysphagia (OD) is a condition recognised by the World Health Organisation and defined as the difficulty or inability to move a bolus safely and effectively from the oral cavity to the oesophagus, and can include aspirations, choking, and residue [1,2]. OD is a pandemic among several phenotypes of older people, affecting between 27% and 91% of the population above 70 years of age, and specifically frail older patients [3]. OD has been recognised as a geriatric syndrome by the European Society for Swallowing Disorders and the European Union Geriatric Medicine Society due to its high prevalence and association with many comorbidities with poor outcomes, such as malnutrition, respiratory infections and aspiration pneumonia, functional disability and frailty, institutionalisation and increased hospital readmissions, and mortality [2].

Compensatory strategies are currently the most common treatment for older patients with swallowing disorders [3]. Two independent reviews found that higher viscosity prevented airway invasion and was therefore effective in managing OD [2,4]. However, these reviews also recommended that (a) thickeners other than starch should be investigated as starch had drawbacks such as residue, poor palatability, and compliance; and (b) further research was necessary to determine the best degrees of viscosity for each phenotype of patient with dysphagia, and to measure and describe them in standardised terminology [4]. The literature also suggests a need to study fluid behaviour in the context of the physiological processes involved in oral preparation, transport, and pharyngeal flow [4]. We identified the oral and pharyngeal processes that can alter the therapeutic effect of thickening products (TPs) and described them using SI units (mPa·s). This protocol has been used by different laboratories which accurately measured ShV with similar results [5]. There is now a new generation of thickeners that are gum-based and presumed to have better protective properties, including leaving less residue than starch-based thickeners [6]. More research needs to be conducted to determine the most effective levels of viscosity to manage older patients with OD [7]. However, most TPs are commercialised using the same qualitative descriptors for very different viscosities once measured in mPa·s [7]. It is important that the labels on TP products state that the viscosity achieved at 50 s^−1^ in SI units (mPa·s at 50 s^−1^) as well as the changes brought about by oral salivary amylase (α-SA) and pharyngeal shear thinning, as both these factors affect the therapeutic action of thickening agents [7].

These agents improve swallowing safety by increasing the viscosity of fluids, and thus avoid aspirations and other complications that could arise [8,9,10]. Viscosity is a rheological property which measures the resistance of a fluid to flow, expressed in SI units as mPa·s [11,12]; rheology is defined as the study of the flow and the deformation of fluids [11,12]. The two major factors that can affect the viscosity of thickened alimentary fluids are salivary α-amylase, during the oral phase of swallow, and shear thinning, which is related to bolus velocity in the pharyngeal phase [7,11]. α-amylase is a salivary enzyme that breaks down starch molecules, the main components of modified-starch thickening agents, and dramatically reduces their viscosity [13]. Shear thinning is a rheological property of non-Newtonian fluids where viscosity is reduced with increases in the bolus velocity and shear rate [7,11]. TPs have a shear thinning behaviour due to the increment in bolus velocity and shear rate during the swallowing process, which ranges from 50 s^−1^ in the oral cavity to 300 s^−1^ in the pharynx. Both salivary α-amylase and shear thinning can decrease the viscosity of the bolus in the oral and pharyngeal phases and therefore increase the risk of aspiration, particularly for modified-starch TPs [7]. Fluid thickening also produces a decrease in pharyngeal bolus velocity at high viscosity levels, and increases the time to upper oesophageal sphincter opening (UESO) in most clinical studies [8,10,13]. In contrast, mechanisms of airway protection such as time to laryngeal vestibule closure (LVC) are generally unaltered [10,14].

Tsururinko Quickly (TQ) is a xanthan gum-based TP, which includes dextrin, calcium lactate, and trisodium citrate in its composition. TQ is manufactured and commercialised in Japan by Morinaga Milk Industry Co. Ltd. The main aim of this study was to assess the safety and efficacy of TQ by videofluoroscopy (VFS) in older patients with severe OD. The second aim was to determine the appropriate shear-viscosity levels for safe and efficient swallowing in older patients with OD, and to characterise the rheological properties in the oral (amylase resistance) and pharyngeal (shear thinning) phases.

## 2. Materials and Methods

### 2.1. Study Population

This clinical trial included 85 patients who were recruited from August 2020 to August 2022 at the Gastrointestinal Physiology Laboratory of the Hospital de Mataró, Consorci Sanitari del Maresme (CSdM), Catalonia, Spain. The study recruitment process was severely delayed due to the COVID-19 pandemic [15].

Inclusion criteria were being more than 70 years old, having clinical signs or symptoms of OD/swallowing dysfunction, the impaired safety of swallow in VFS study (PA ≥ S3), and giving written informed consent. Exclusion criteria were patients with safe swallowing in a VFS study (PAS 1 or 2), an OD associated with structural alterations (such as osteophytes, Zenker diverticulum, bars), dementia and severe cognitive disorders causing an inability to comply with the protocol requirements, pregnancy or lactation, and allergy to any study ingredient. Accepting an alpha risk of 0.05 and a beta risk of 0.1 in a two-sided test, 83 subjects are necessary to recognise a difference in safe swallowing prevalence as statistically significant, calculated with the interim analysis data (n = 60) consisting in an initial proportion of 0.15 at <50 mPa·s, and a final proportion of 0.62 at 100 mPa·s. An estimated drop-out rate of 63.50% was determined.

The study protocol, informed consent form, and information given to the participants were approved by the Ethics Committee of the CSdM under the code 59/18. The study was conducted according to the principles of the ‘World Medical Association Declaration of Helsinki’ (2013) and the International Conference on Harmonization (ICH) Guidelines for Good Clinical Practice (GCP, September 1997), as appropriate for Spanish nutritional products legislation where the study took place. This study was registered in the Clinical Trials Gov website with code: NCT04565587.

### 2.2. Material and Methods

#### 2.2.1. Products

The TP used for the study was Tsururinko Quickly (TQ), manufactured by Morinaga Milk Industry, Co., ltd, Tokyo, Japan. This TP is composed of xanthan gum, dextrin, calcium lactate, and trisodium citrate containing the following nutrients (per 100 g): 270 kcal, 0.5 g protein, 88.90 g carbohydrates, 21.90 g fibre, 960 g sodium, 980 g potassium, 30 mg phosphorus, 4.50 g ash, and 6.10 g water. The mineral water used for the study was Font D’Or manufactured by Vichy Catalan Corporation, Barcelona, Spain. The X-ray contrast used for VFS studies was Omnipaque™ commercialised by GE Healthcare Bio-Sciences, S.A.U, Madrid, Spain.

The swallowing of boluses at 6 shear viscosity levels (<50, 100, 200, 400, 800, and 1600 mPa·s) was examined by VFS following a standardised preparation protocol [5]. For VFS, the solution was prepared according to 50 mL 1:1 Omnipaque and water. For rheological tests, two viscosities (200 and 800 mPa·s) were selected. Solutions were prepared with 100 mL mineral water. TP doses to prepare each viscosity level are presented in Table 1.

#### 2.2.2. Equipment

In the videofluoroscopy (VFS) studies, images were recorded at 25/s with a Panasonic AG DVX-100B (Matsuhita Electric Industrial Co, Ltd., Osaka, Japan) and recordings were obtained with a Super XT-20 Toshiba Intensifier (Toshiba Medical Systems Europe, Zoetermeer, The Netherlands). Patients’ swallows and their oropharyngeal swallowing response (OSR) were analysed with the software Swallowing Observer (Image & Physiology SL, Barcelona, Spain) as previously described in our laboratory.

In the rheological studies, shear viscosity was measured with a rotational viscometer (Haake Viscotester^®^ 550, Thermo Fisher Scientific, Waltham, MA, USA). For viscosities between 50 and 300 mPa·s, the MV1 rotor was selected; for higher viscosities, the SV1 rotor was used. Temperature was maintained at 25 °C by the ThermoScientific system. Ten microliters (10 mL) of each sample was introduced into the sensor system and the resistance of the bolus to the rotational movement produced by the rotor was measured in a shear rate range from 1 to 1000 s^−1^. Results were analysed by the software RheoWin 4.60.0000 (Job Manager^®^ and Data Manager^®^) as previously described [7].

### 2.3. Experimental Design

This was an interventional, non-randomised, multiple doses, fixed-order, and single-centre study to analyse the therapeutic effect of a thickening product with VFS in older patients suffering from severe OD due to the impaired safety of swallowing. The overall study procedure was performed in a single visit. Older patients with OD who fulfilled the inclusion criteria were included in this study. Then, 48 h after the VFS study, patients were asked by telephone about any potential adverse event (AE) occurring after the test.

#### 2.3.1. Videofluoroscopy

**Figure 1:**
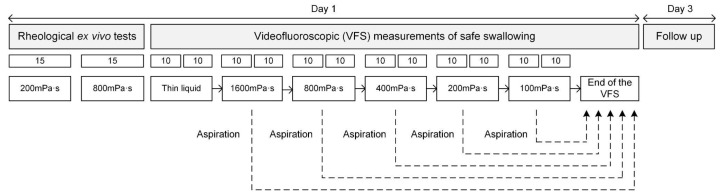
Experimental design of TQ study NCT04565587 at Consorci Sanitari del Maresme.

Patients were imaged while swallowing bolus viscosities seated in a lateral projection which included the oral and pharyngeal cavity and proximal oesophagus [13,16]. During the VFS, patients were asked to swallow 10 mL boluses per duplicate for each viscosity level following the safety algorithm shown in Figure 1. Briefly, the procedure commences with liquid boluses followed by the highest viscosity bolus to the lowest. A safety rule dictates that the study is discontinued if the patient aspirates while swallowing any of the thickened boluses.

VFS sequences were analysed to determine the visuoperceptual signs of impaired safety (safe swallows, mean PAS score, penetrations, and aspirations), those of the impaired efficacy of swallowing (oral and pharyngeal residue), and the severity of the oral and pharyngeal residue (0 = no residue, 1 = coating residue, 2 = pooling residue) [17]. We also assessed the biomechanics of the oropharyngeal swallow response (OSR), including the time to laryngeal vestibule closure (LVC), upper oesophageal sphincter opening (UESO), and the kinematics of swallowing (mean and final bolus velocity in the pharynx and bolus kinetic energy prior to entering the UESO) [13].

#### 2.3.2. Rheological Ex Vivo Characterisation

Two major rheological properties were analysed according to a previously defined protocol [5]: the effect of α-salivary amylase on viscosity in the oral phase and the effect of bolus velocity in the pharyngeal phase (shear thinning), as well as the combined effect of both factors in older patients with OD. For the assessment of the rheological properties of the TP, a pre- and post-oral incubation viscosity analysis was performed on every patient. All participants held a bolus of 15 mL of each viscosity in the mouth for 30 s and then spat it out to be analysed by the viscometer. These ex vivo tests were always performed in the morning to avoid variations in saliva volume or salivary amylase concentration due to the circadian cycle of saliva secretion.

All samples were analysed by triplicate in the viscometer with a constant temperature of 25 °C, and the mean was calculated. Viscosity at 50 and 300 s^−1^ was extrapolated from the regression line of the means of the three measurements obtained from the shear rate range from 0 to 1000 s^−1^. The viscosity flow curve was adapted to the Ostwald de-Waele model (power law) as previously described [7].

The shear rate and α-salivary amylase effect on viscosity were only determined for 200 and 800 mPa·s viscosity levels to avoid further inconveniences to the participants. A reduction in viscosity caused by the shear rate (viscosity at 300 s^−1^) and salivary amylase (viscosity at 50 s^−1^ post-oral incubation) was calculated as the obtained difference in viscosity, taking viscosity at 50 s^−1^ previous to the oral incubation as a reference value.

#### 2.3.3. Hedonic Scale

During VFS, and after each swallow, the study participants were asked for their opinion on the palatability of the product with the 5-point facial Likert hedonic scale, rating how much they enjoyed it on a scale from 0 to 5.

#### 2.3.4. Adverse Events

The Common Terminology Criteria for Adverse Events (CTCAE; U.S. Department of Health and Human Services, National Institutes of Health v5.0; Gastrointestinal Disorders) were used to score the severity of adverse events. To measure the relation between the product and any adverse event, the World Health Organisation (WHO) and the Uppsala Monitoring Centre (UMC) for standardised causality assessment system was used [18].

#### 2.3.5. Outcome Parameters

In terms of the main variable, the percentage (prevalence) of participants that swallowed safely (PAS score 1, 2) for each viscosity level [19] was recorded.

In terms of the secondary variables, the safety of swallowing was measured by mean PAS score, recording the percentage of patients that safely swallowed (PAS score 1, 2); the percentage of patients with bolus penetration into the laryngeal vestibule (PAS scores of 3, 4, and 5); the percentage of patients with aspiration into the airway (PAS scores of 6, 7, and 8); and the comparison between each level of viscosity [19]; the efficacy of swallowing was expressed by the prevalence and severity of the oral residue and pharyngeal residue (0, 1, 2) [17] and the effect of oral incubation at 50 s^−1^ with salivary amylase and the additional effect of pharyngeal shear thinning at 300 s^−1^ for 200 and 800 mPa·s viscosity levels.

### 2.4. Data Analysis

Quantitative data were described as the means ± standard deviation (SD). The statistical test applied for oral motor response and the kinematics of swallowing was the non-parametric ANOVA test (Kruskal–Wallis) with Dunns’ multiple test to compare all groups. Qualitative data were described as a percentage of absolute frequencies including the safety and efficacy of swallowing. The statistical test applied was the chi-square for global comparison and Fishers’ test for 1:1 difference. Significance was considered at *p* < 0.05. All statistical analysis were performed with GraphPad Prism 6.0.

## 3. Results

### 3.1. Demographics and Patient’s Clinical Characteristics

A total of 305 patients were screened and participated in the VFS study. Among these, 91 participants were excluded for presenting OD structural causes (29.83%, mainly osteophytes or CP bars), and another 105 patients were excluded because they presented safe swallowing with PAS 1 and 2 (34.42%); however, 2.9% of recruited patients were not able to perform the VFS procedure and were excluded due to a protocol deviation or interruption such as nausea or the inability to complete the series of boluses or stay seated while performing the VFS, and 4.3% of patients were withdrawn due to technical problems during the study. Finally, 85 patients with OD and VFS signs of impaired safety of swallow, PAS ≥ 3 completed the study.

**Figure 2:**
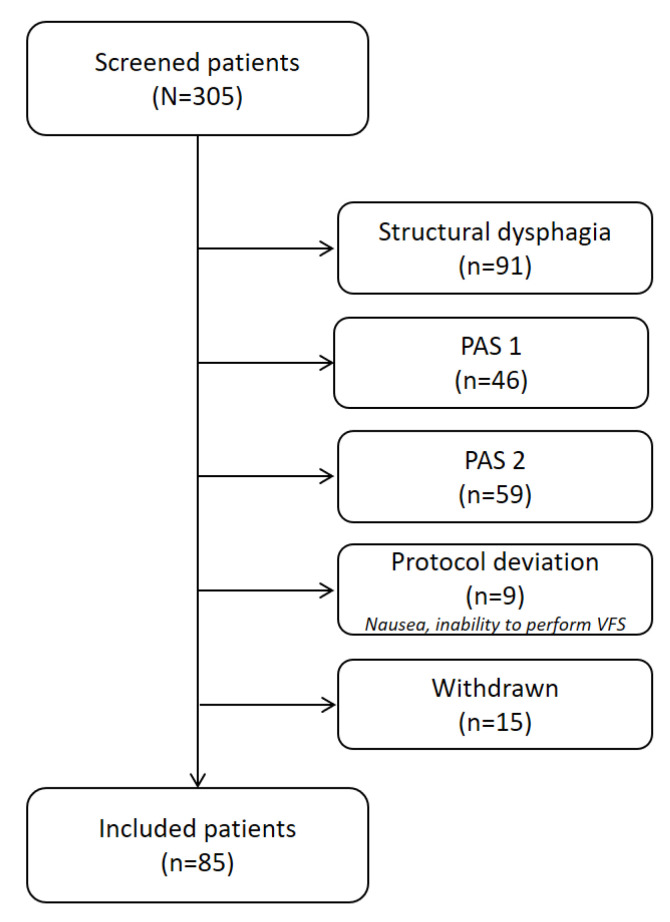
Consort flow chart of patient recruitment and inclusion in the study. PAS indicates the penetration–aspiration scale.

The mean age of the study population was 83 ± 6.93 years and 53.33% were male. Regarding OD causes, 45% were only associated with ageing, 36.67% of patients had suffered from a stroke in the past, and 18.33% presented neurodegenerative diseases such as Parkinson’s or Alzheimer’s disease (Figure 2).

### 3.2. Therapeutic Effect

#### 3.2.1. Safety of Swallowing

**Figure 3:**
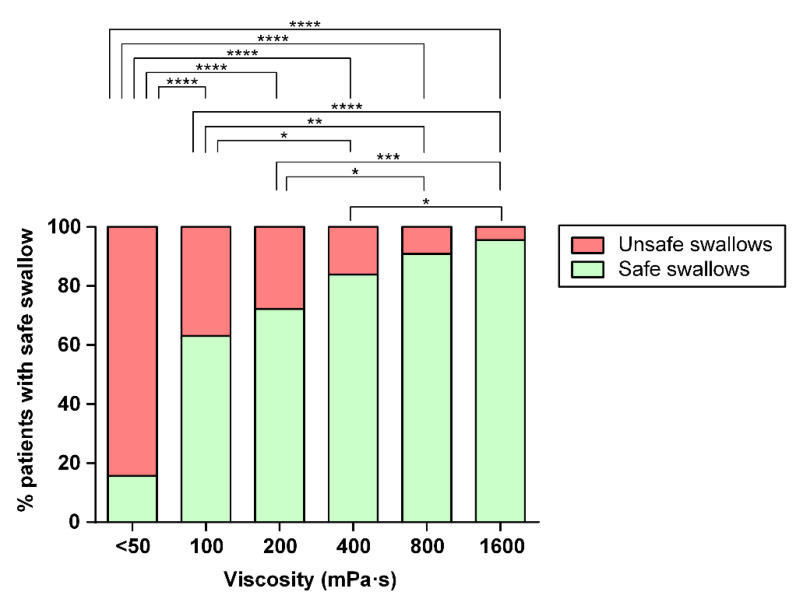
Prevalence of patients with safe swallowing at each viscosity level assessed herein. Significant differences are also presented for all viscosities. * *p* < 0.05; ** *p* < 0.01; *** *p* < 0.001; **** *p* < 0.0001.

Up to 83.75% of the older patients with OD included in this study presented unsafe swallowing with thin liquid; 45.00% (PAS 3–5) of whom were patients with penetrations and 38.75% with aspirations (PAS 6–8). The fluid thickening with TQ had a strong viscosity-dependent therapeutic effect on the prevalence of patients with safe swallowing ranging from 62.90% at 100 mPa·s to 95.24% at 1600 mPa·s. Differences were significant for all the thickened levels vs. thin liquid (*p* < 0.0001), as shown in Figure 3. The prevalence of patients with safe swallowing at each viscosity level is also presented in Table 2. The PAS value at each viscosity level is presented in Supplementary Figure S1. The number of patients studied at each viscosity level is also included.

**Figure 4:**
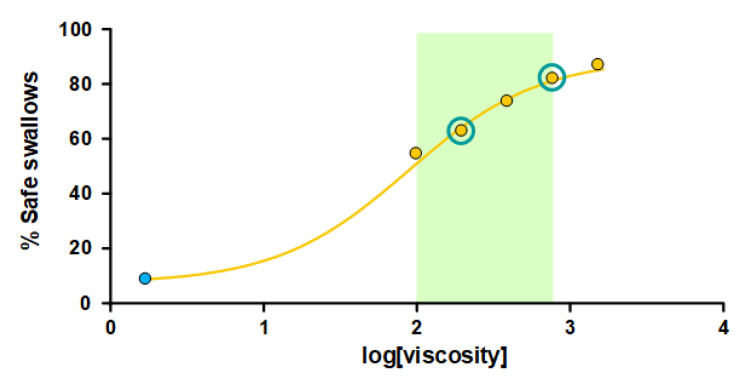
Dose–response curve. The effect of increasing viscosity on the prevalence of patients with safe swallowing. The green frame depicts the therapeutic range of TQ in older patients with OD. The green circles represent the two optimal viscosity levels needed to ensure safe swallowing for all the patients included in this study.

Increasing the shear viscosity strongly reduced the prevalence of patients with penetrations or aspirations. Penetrations were reduced in a viscosity-dependent manner by 30.65% at the 100 mPa·s viscosity level and 3.57% at 1600 mPa·s. Aspirations were also reduced from 11.43% at 200 mPa·s to 1.19% at 1600 mPa·s. The prevalence of patients with penetrations and aspirations and a mean PAS score for each viscosity level is presented for all viscosity levels in Table 2. The therapeutic range for TQ was determined between 100 and 800 mPa·s. The threshold effect for safety of swallowing was observed at 100 mPa·s and the maximal therapeutic effect was observed at 800 mPa·s with a prevalence of patients with safe swallows of 90.36% and no significant differences with the highest viscosity level assessed herein (1600 mPa·s). The optimal viscosity levels to treat this population of significantly older patients with severe OD and an impaired safety of swallowing with this specific TP were 200 and 800 mPa·s (Figure 4). The lower level was selected for presenting no significant differences vs. the following higher level (400 mPa·s) regarding safe swallowing and for reducing penetrations (PAS 3–5) in a significant manner (*p* < 0.001) when compared to the <50 mPa·s viscosity level (thin liquid).

#### 3.2.2. Efficacy of Swallowing

No viscosity-dependent effect was observed on the oral or pharyngeal residue (Supplementary Figure S2). The oral residue significantly increased at 200 (71.43% of patients; *p* = 0.003), 400 (68.35%; *p* = 0.006), and 1600 mPa·s (69.05%; *p* = 0.004) when compared to the thin liquid (46.25%). In contrast, pharyngeal residue varied very little between the viscosity levels, ranging from 11.25% to 20.24%. The coating residue according to the Robbins’ scale was the most prevalent in both the oral and pharyngeal areas. The prevalences of the coating and pooling residues are shown in Table 2 and in Supplementary Figure S3.

#### 3.2.3. Oropharyngeal Swallowing Response

**Figure 5:**
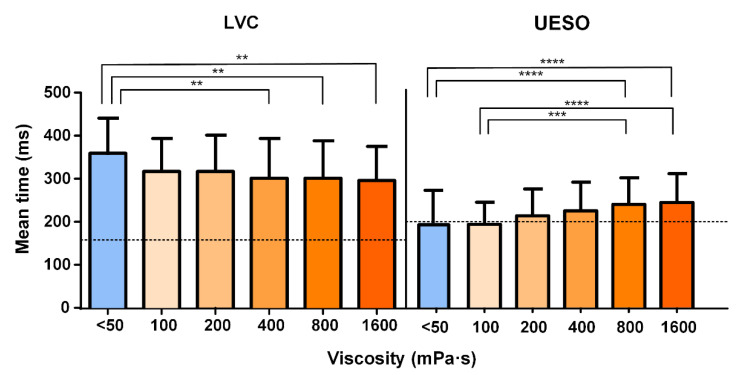
Effect of the bolus velocity on the oropharyngeal swallow response. The mean times to laryngeal vestibule closure (LVC) and upper oesophageal sphincter opening (UESO) for each viscosity level assessed herein are shown. The dashed line represents the reference value in healthy volunteers (160 ms for LVC according to Clavé 2006; 200 ms for UESO according to Rofes 2010). ** *p* < 0.01; *** *p* < 0.001; **** *p* < 0.0001.

1. For the airway protection mechanism, the time to LVC was reduced by increasing the shear viscosity from 360 ± 90.18 at <50 mPa·s to 300 ± 84.16 ms at 1600 mPa·s (Figure 5).
2. For the bolus transit, the time to UESO was moderately increased by thickening the fluid, showing significant differences for <50 mPa·s vs. 800 and 1600 mPa·s (*p* < 0.0001). The mean UESO is represented in Figure 5 with the statistically significant differences obtained for all viscosity levels. The times to LVC and UESO values are presented in Table 3.
3. For the kinematics of swallowing, the mean bolus velocity was reduced by increasing the bolus viscosity ≥ 800 mPa·s, ranging from 0.33 ± 0.18 to 0.22 ± 0.08 m/s. Significant differences appeared for <50, 100, and 200 mPa·s vs. 800 and 1600 mPa·s (Supplementary Figure S4). The bolus kinetic energy went from 1.29 ± 1.04 to 3.14 ± 6.00 mJ. Significant differences appeared for <50 and 100 mPa·s when compared to 800 and 1600 mPa·s. Values for the kinematics of swallowing are presented in Table 3.

### 3.3. Adverse Events

Adverse events’ reports were obtained from 71.76% of the participants (n = 61). A total of 22.95% (n = 14) of these patients presented adverse events: diarrhoea (19.67%; n = 12), abdominal pain (1.63%; n = 1), and nausea (1.63%; n = 1). All of them were classified as unlikely to be related to the study product as gastrointestinal disorders are reported in the technical sheet of the X-ray contrast. No serious adverse events were reported during or following this study.

### 3.4. Hedonic Scale

**Figure 6:**
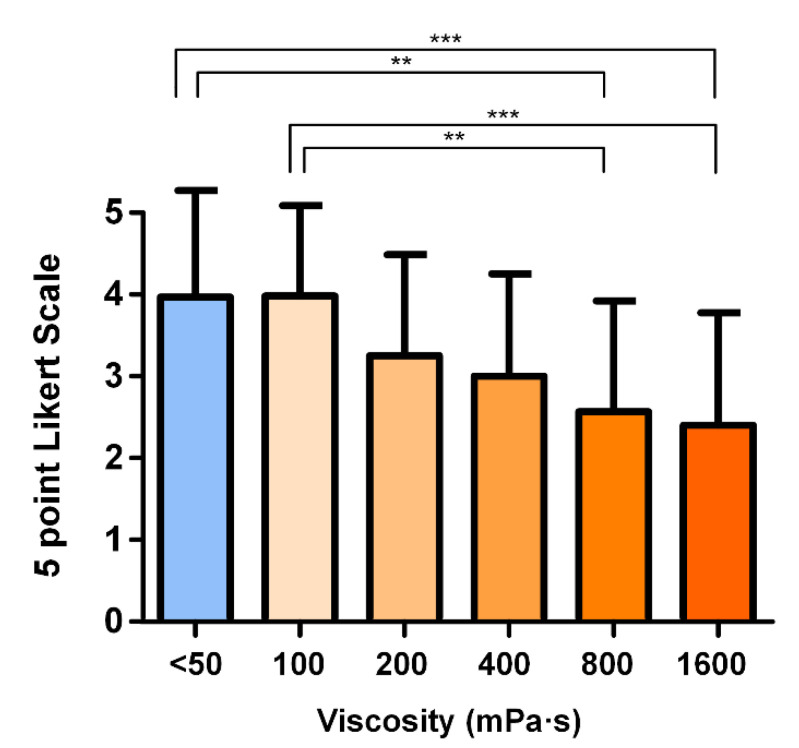
Mean ± SD punctuation on palatability given by the participants according to the 5-point facial Likert scale at each viscosity level assessed. ** *p* < 0.01; *** *p* < 0.001.

The mean punctuations for each viscosity level assessed herein are as follows: 3.93 ± 1.34, 3.96 ± 1.08, 3.25 ± 1.24, 3.00 ± 1.25, 2.57 ± 1.36, and 2.4 ± 1.38 from thin liquid to 1600 mPa·s, respectively. The palatability decreased in a viscosity-dependent manner. However, significant differences only appeared for the highest viscosity levels assessed (800 and 1600 mPa·s) vs. thin liquid (<50 mPa·s) and vs. 100 mPa·s (Figure 6).

### 3.5. Rheological Ex Vivo Characterisation

**Figure 7:**
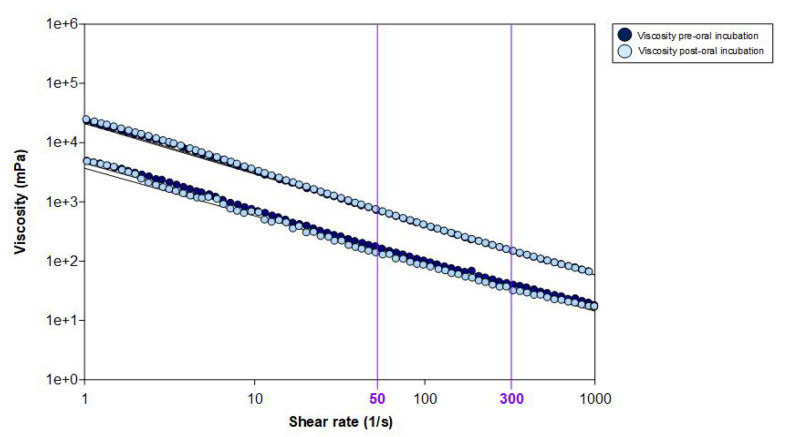
Viscosity flow curves of the pre- and post-oral incubation viscosity levels of 200 and 800 mPa·s, respectively, in a shear rate range from 1 to 1000 s^−1^.

The measurements of shear viscosity at 50 s^−1^ were 189.65 ± 2.39 and 768.90 ± 19.76 for the 200 and 800 target viscosity levels, respectively. After oral incubation in 84.71% of the study participants (n = 72), we observed a non-significant decrease in the viscosity of 17.18% (157.07 ± 49.75 mPa·s) for 200 mPa·s, and an increase of 2.01% for 800 mPa·s (784.38 ± 99.84 mPa·s) clearly showing an amylase resistance of TQ. Shear thinning at the pharyngeal shear rate produced a mean decrease in viscosity of 77.25% at 300 s^−1^. Both swallowing factors (oral incubation and pharyngeal shear thinning) caused a global shear-viscosity decrease of 78.96%. Figure 7 shows the viscosity flow curve of shear viscosity for the pre- and post-oral incubation viscosity levels of 200 and 800 mPa·s, respectively.

## 4. Discussion

The main result of this study was that fluid thickening with TQ in a therapeutic range of 100–800 mPa·s strongly increased the safety of swallowing in older patients with severe OD in a viscosity-dependent manner without any significant increase in the prevalence of pharyngeal residue. Regarding its mechanisms of action, at low viscosity levels (100–200 mPa·s), the therapeutic effect of TQ relies on its intrinsic rheological properties; at viscosities of ≥400 mPa·s, the time to LVC improves, and at viscosities ≥800 mPa·s, the bolus velocity is also delayed. Our study shows that TQ is unaffected by oral salivary amylase and is well accepted and tolerated in older patients with OD. Our study clearly shows that therapeutic strategies and interventions using fluid thickening with TQ at the two optimal viscosity levels of 200 and 800 mPa·s can provide safe and efficient swallowing in most older patients with OD and an impaired safety of swallowing.

Ageing is the main demographic change in developed countries and the prevalence of older people with OD is growing and becoming a new pandemic. It has been estimated that 16 million American (USA), 30 million European, and 8 million Japanese citizens have age-related OD and are at increased risk of aspiration pneumonia, malnutrition and dehydration, functional disability, frailty, hospitalisation and institutionalisation, and poor clinical outcomes [3,20]. We have previously studied the same phenotype of older patients with an impaired safety of swallowing [21]. We enrolled 1662 patients from the acute geriatric unit and found that 47.4% had OD and 30.6% had malnutrition. Both conditions were associated with multimorbidity, multiple geriatric syndromes, and poor functional capacity, as well as increased intrahospital, 6-month, and 1-year mortality rates [21]. In another study, OD was a prevalent risk factor associated with hospital readmission for both aspiration and non-aspiration pneumonia in the very old [20]. Finally, more recently, we proved OD also causes high economic costs during hospitalisation that significantly increase in the case of malnutrition and respiratory infections at long-term follow-up [22,23] and severely impact the quality of life of these older citizens.

This growing population of older people with OD, especially those with an impaired safety of swallowing, has new specific needs regarding fluid-thickening and texture-modified foods tailored to their swallowing and chewing disabilities. Treatment for OD in older people is mainly based on compensating swallowing impairments through increasing fluid viscosity and adapting food textures to avoid aspiration, choking, and respiratory infection, and improving nutritional status, hydration, and oral health. This has been defined as the minimal-massive intervention, the minimal effective treatment to be provided to all members of this significantly older population, and TPs are key elements in these evidence-based, multimodal interventions to provide safe swallowing and hydration [9]. The main requirements for the new generation of TPs should be that they include a strong therapeutic effect at low shear viscosity levels, minimal residue, high palatability to guarantee treatment compliance, evidence-based optimal viscosity levels for this phenotype of patients, and appropriate labelling and terminology—including measurements of their rheological properties in SI units. The design of the present clinical trial aimed to answer all these questions for TQ, and to the best of our knowledge, this is the first study to use objective data from VFS to explore the therapeutic effect of six levels of shear viscosity of this modern XG-based TP in older patients with severe OD, an impaired safety of swallowing, and a high prevalence of aspirations.

Our study clearly shows that the safety of swallowing in these older patients with severe OD and aspirations can be significantly and dramatically improved by increasing the viscosity with TQ using the minimal thickening level assessed herein (100 mPa·s). The therapeutic effect frame and the assessment of the maximal significant shear viscosity level (800 mPa·s) to manage patients with OD was also determined by a viscosity-response effect that shows that there is no further significant improvements in the safety of swallowing above 800 mPa·s, and that, by only using only two shear-viscosity levels (200 and 800 mPa·s) in these multimodal treatment protocols, it is possible to provide safe swallowing in almost all older patients (90%) with OD by means of TQ. We selected 200 mPa·s as the minimal optimal dose to manage patients with a lower severity of dysphagia due to that fact that: (a) they presented significant differences for safely swallowing when compared to thin liquid; (b) they did not present significant differences in safely swallowing when compared to 400 mPa·s; and (c) because there is a significant difference in penetration between the thin fluid and 200 mPa·s, but not between the thin fluid and 100 mPa·s. The maximal optimal dose (800 mPa·s) was chosen because: (a) it was shown to provoke significantly less aspirations than 400 mPa·s (2.42% vs. 10.13%, *p* = 0.053), and; (b) it did not present significant differences in terms of safe swallowing when compared to 1600 mPa·s.

The shear-viscosity-dependent effect of several types of TP on the safety and efficacy of swallowing in older patients with OD were studied in previous clinical trials in similar and different phenotypes of patients. A study published by our group in 2020 also including older patients with OD (82.96 ± 1.24 y) showed a therapeutic range for a mixed TP between 250 and 1000 mPa·s [10] and no significant improvement in the safety of swallowing were found between 1000 mPa·s and the highest viscosity level assessed, which was 2000 mPa·s. A similar result was observed for post-stroke and Parkinson patients with OD in the same study. In contrast, patients with HNC had a more severe OD and increasing shear viscosity within the same therapeutic range had a lesser effect [10]. Another recent study also developed in our laboratory with an XG TP in patients with chronic post-stroke OD and a mean age of 76.7 ± 8.9 years found that the therapeutic range for these patients was 150–800 mPa·s; 150 mPa·s was the lowest viscosity to significantly improve the swallowing safety and viscosities above 800 (such as 1400 and 2000 mPa·s), and did not significantly improve swallowing safety in post-stroke patients with OD [8].

Our present study suggests that 800 mPa·s is the maximal shear viscosity level required to treat the most severe older patients with TQ. Some countries such as the US (NDD), Japan (JDD2021), and Australia have their own standards for managing dysphagia. We evaluated each country’s standards based on our results. We felt that “Spoon-thick” for NDD standards (>1750 mPa·s) was an excessively high viscosity and “Honey-like” (351–1750 mPa·s) was an excessively wide viscosity range. Also, the maximum management range for JDD2021-‘standards was 500 mPa·s, but it may be better to raise the upper limit to 800 mPa·s if all severe patients are to be managed using only TP (or high-viscosity liquid foods) [24]. “Extremely thick” in the Australian standard was 900 mPa·s and above, which is close to our conclusion [25,26]. We also believe that textural classifications that only use qualitative descriptors for viscosity levels such as IDDSI are too broad and inaccurate to prescribe these specific levels of viscosity. TP labels must include viscosity in SI units (mPa⋅s at 50 s^−1^) and not in qualitative descriptors, to accurately identify and select these specific levels of shear-viscosity [7], and that the dose and preparation method must be described on the label of the TP [7]. Regarding the swallowing efficacy, TQ caused a moderate increase in the coating of oral residue at almost all viscosity levels. In contrast, the effect on the pharyngeal residue was minimal and not significant. Pharyngeal residue has been related to an increased risk of post-swallowing aspirations and it is a significant negative effect when using MS thickeners at high viscosity levels and a clear advantage of the newest generation of XG TPs [26,27]. We previously found that older patients with OD present unsafe swallowing and slow swallowing biomechanics, as well as pharyngeal hypoesthesia with the disrupted conduction of pharyngeal sensory inputs [15,28]. The cut-off to predict unsafe swallows with time to LVC was established at 340 ms in older and in post-stroke patients [8,14]. In 2020, our group published the results on a study including four phenotypes of patients with OD (post-stroke, older, neurodegenerative diseases, and head and neck cancer), and all of them reported a severely delayed time to LVC ranging from 360 to 428 ms [10]. A clinical trial performed on post-stroke OD patients also presented the results in line with these values where the recruited population reported a time to LVC above 380 ms. Our present study shows a severe impairment in the airway protection mechanisms in these older patients with OD as the mean time to LVC (380 ms) was strongly delayed in all the study population, thus indicating a significant risk of aspirations.

In the present study, we also explored the mechanisms causing the therapeutic effect of TQ. The increment in shear viscosity produced significant variations in this specific airway protection mechanism (time to LVC). In contrast, the time to UESO was significantly delayed as the shear viscosity was increased and mean velocity was also significantly reduced at 800 and 1600 mPa·s. From these results, we observe that the therapeutic effect of TQ on the safety of swallowing TQ is due to three major sequential mechanisms: (a) the intrinsic rheological characteristics of TQ at 100–200 mPa·s, as there are no changes in the biomechanics of the swallowing response; (b) to an improvement in the airway protection mechanisms by reducing the time to LVC at 400 mPa·s and the viscosity levels above; and (c) and a kinematic effect for viscosities of 800 mPa·s and above by reducing the bolus velocity, which also increases the time to UESO. In addition, although XG TP solutions are liquids, from a rheological point of view, they have a ‘yield stress’, which is a property of solids or gels [29]. Yield stress is the stress at which the flow begins and is related to the degree of internal binding force [30]. Yield stress can therefore be considered an index of the cohesiveness of a food bolus. As a non-invasive biometric, acoustic analysis was performed [29,30], showing that the time required for the food bolus to pass through the pharyngeal phase decreases with an increasing concentration of the XG TP solution. The preference for non-Newtonian fluids for safe swallowing should be influenced not only by the shear rate dependency, but also by elasticity to provide mechanical cohesiveness with a food bolus [30].

Finally, it is well known that increased viscosity decreases the palatability of TPs. This is one of the main limitations to the patient acceptance of fluid thickening and therapeutic compliance. It is also confirmed by the results of this study which show that significant differences were appreciated by participants for the most thickened levels tested herein (800 and 1600 mPa·s). In addition, boluses were prepared for an acute situation (with X-ray contrast) which does not reflect the daily life situation of the patients. However, palatability seems of little importance when prescribing a TP but patients’ needs must be understood to prevent non-compliance with the prescribed treatment. Another limitation of this study arises from the safety rule used to protect participants from repetitive aspirations. Due to this measure, not all participants received all the viscosity levels selected (especially the lowest levels 100–200 mPa·s). Participants included in this study correspond to different OD phenotypes which might present some differences in the swallowing response. Overall, this study reflects the situation we found in standard clinical practice, representing the population of older patients with oropharyngeal dysphagia we manage in a dysphagia unit.

## Figures and Tables

**Table 1:** Doses used to achieve each viscosity level for the videofluoroscopy and rheological tests.

Target Viscosity (mPa·s) at 50 s^−1^	Tsururinko Quickly (g)	Final Volume (mL)	Dissolvent (mL)
**VFS doses**			
<50	-	50	1:1 (water:Omnipaque)
100	0.58	50	1:1 (water:Omnipaque)
200	1	50	1:1 (water:Omnipaque)
400	1.45	50	1:1 (water:Omnipaque)
800	2.45	50	1:1 (water:Omnipaque)
1600	4.3	50	1:1 (water:Omnipaque)
**Rheological test doses**			
200	2	100	Water
800	5.8	100	Water

VFS: videofluoroscopy.

**Table 2:** Prevalence of safe swallows, penetrations, aspirations, oral, and pharyngeal residue at each viscosity level assessed. Safe swallows = PAS 1, 2; penetrations = PAS 3–5, aspirations = PAS 6–8.

Prevalence (%)	Viscosity Level (mPa·s)					
	<50	100	200	400	800	1600
Safe swallows	16.25	62.90	71.43	82.28	90.36	95.24
Penetrations	45.00	30.65	17.14	7.60	7.23	3.57
Aspirations	38.75	6.45	11.43	10.13	2.41	1.19
Mean PAS ± SD	4.91 ± 2.16	2.55 ± 1.87	2.47 ± 1.92	2.11 ± 1.97	1.53 ± 1.14	1.31 ± 0.92
Oral residue *Coating* *Pooling*	46.25	58.06	71.43	68.35	65.06	69.05
	41.25	50.00	64.29	59.49	49.40	52.38
	5.00	8.07	7.14	8.86	15.66	16.67
Pharyngeal residue *Coating* *Pooling*	11.25	11.29	14.29	12.66	16.87	20.24
	8.75	8.07	11.43	8.86	13.25	14.29
	2.50	3.23	2.86	3.80	3.61	5.95

**Table 3:** The timing of the airway protection mechanisms (laryngeal vestibule closure) and bolus transfer events (upper oesophageal sphincter opening), mean bolus velocity in the pharynx, final velocity prior to entering the upper oesophageal sphincter, and the bolus kinetic energy at each viscosity level assessed herein.

OSR and Kinematics	Viscosity Level (mPa·s)					
	<50	100	200	400	800	1600
LVC (ms)	360 ± 90.18	323 ± 76.67	324 ± 86.40	305 ± 93.85	307 ± 89.16	300 ± 84.16
UESO (ms)	186 ± 78.20	188 ± 50.35	208 ± 61.54	214 ± 66.85	231 ± 59.60	236 ± 65.60
Mean velocity (m/s)	0.34 ± 0.23	0.28 ± 0.11	0.27 ± 0.09	0.27 ± 0.15	0.23 ± 0.08	0.23 ± 0.10
KE (mJ)	3.14 ± 6.00	2.01 ± 2.43	1.76 ± 1.30	1.75 ± 1.60	1.29 ± 1.04	1.39 ± 1.48

## Data Availability

The data presented in this study are available on request from the corresponding author. The data are not publicly available due to ethical restrictions.

## Supplementary Materials

The following supporting information can be downloaded at: https://www.mdpi.com/article/10.3390/nu15143279/s1, Figure S1: Prevalence of the penetration–aspiration scale (PAS) levels at each viscosity assessed herein. The number of patients assessed at each viscosity level (sample size) is also depicted; Figure S2: Prevalence of patients with oral or pharyngeal residues at each viscosity level assessed. Figure S3: Effect of increasing the viscosity on the prevalence of patients with oral and pharyngeal residue according to the Robins scale (coating vs. pooling). The stripped frame marks pool residue. * *p* < 0.05; ** *p* < 0.01; Figure S4: The mean bolus velocity and kinetic energy for each viscosity level assessed. * *p* < 0.05; ** *p* < 0.01; *** *p* < 0.001; **** *p* < 0.0001.
