# Supplementary material for: Shear-Viscosity-Dependent Effect of a Gum-Based Thickening Product on the Safety of Swallowing in Older Patients with Severe Oropharyngeal Dysphagia
Source: Nutrients. 2023 Jul 24;15(14):3279. doi: 10.3390/nu15143279 (PMC10384341; doi:10.3390/nu15143279)
Supplement: Supplementary file 1 [file nutrients-15-03279-s001.zip › nutrients-2478172-supplementary.pdf]

Supplementary figures

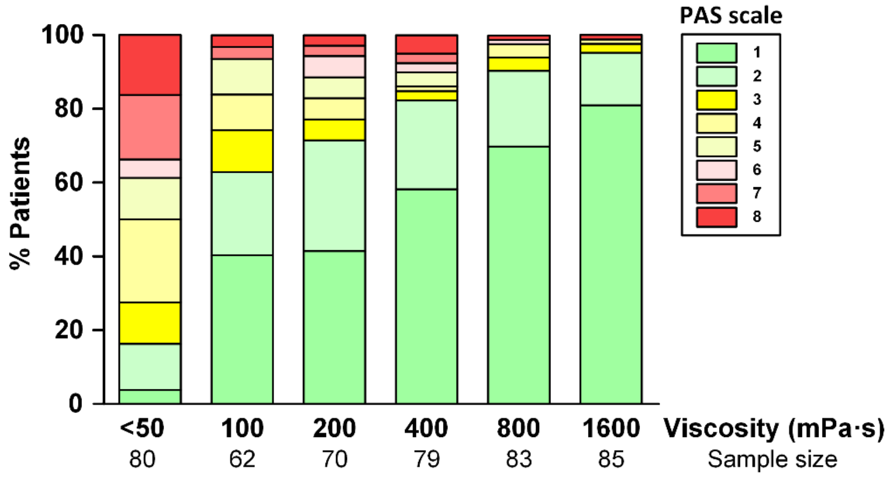

Supplementary Figure S1. Prevalence of Penetration Aspiration Scale (PAS) levels at each viscosity assessed. The number of patients assessed at each viscosity level (sample size) is also depicted.

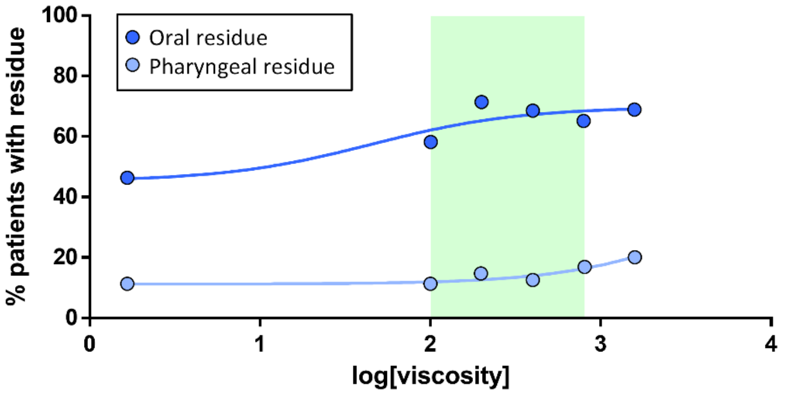

Supplementary Figure S2. Prevalence of patients with oral or pharyngeal residue at each viscosity level assessed.

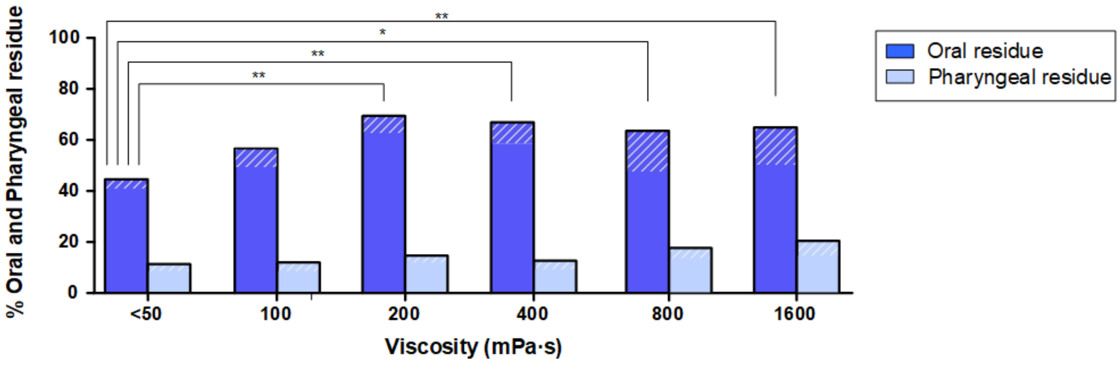

Supplementary Figure S3. Effect of increasing viscosity on the prevalence of patients with oral and pharyngeal residue according to Robins Scale (Coating vs Pooling). The stripped frame marks pool residue. **p<0.05; **p<0.01.*

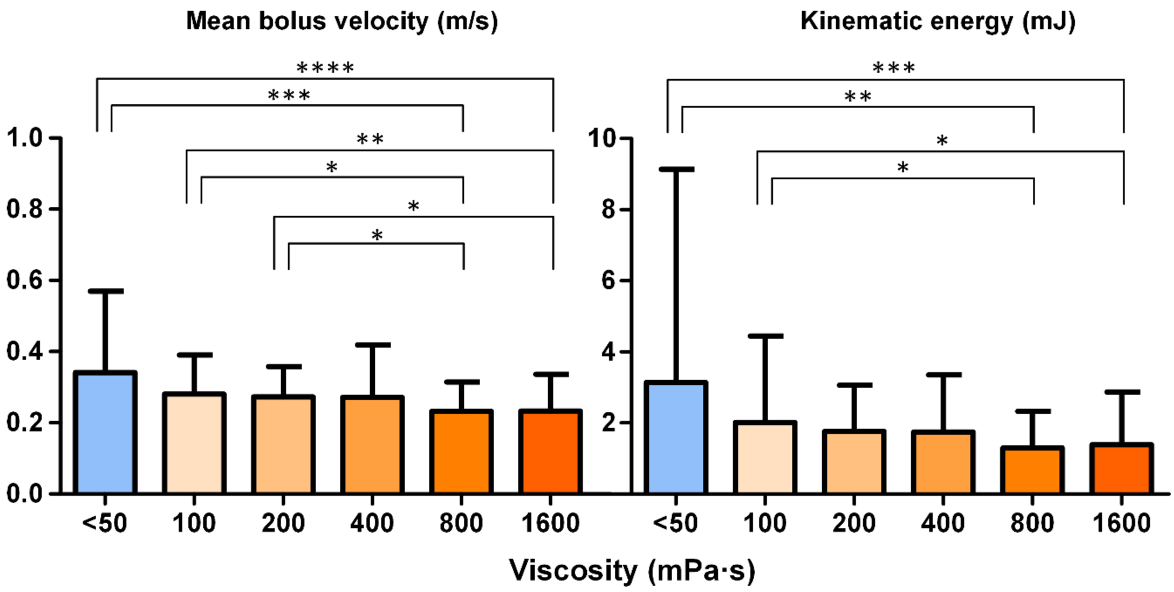

Supplementary Figure S4. Mean bolus velocity and kinetic energy for each viscosity level assessed. *p<0.05; **p<0.01; ***p<0.001; ****p<0.0001.
